# Coupled Hydrological and Biogeochemical Forcings Structure Phytoplankton Community Assembly in a Eutrophic Estuary

**DOI:** 10.3390/microorganisms14061363

**Published:** 2026-06-18

**Authors:** Liang-Gen Wang, Peng-Bing Pei, Tang-Cheng Li, Xiu-Li Yan, Fei-Yan Du, Hong Du

**Affiliations:** 1 Guangdong Provincial Key Laboratory of Marine Biotechnology, Marine Biology Institute, Shantou University, Shantou 515063, China; lgwang@scsfri.ac.cn (L.-G.W.); tcli@stu.edu.cn (T.-C.L.); xlyan@stu.edu.cn (X.-L.Y.); 2 Guangdong Provincial Key Laboratory of Fishery Ecology and Environment, South China Sea Fisheries Research Institute, Chinese Academy of Fishery Sciences, Guangzhou 510300, China; feiyanegg@163.com; 3 Scientific Observing Experimental Station of South China Sea Fishery Resources and Environment, Ministry of Agriculture and Rural Affairs, Guangzhou 510300, China; 4 Guangdong Shantou Ecological and Environmental Monitoring Center, Shantou 515041, China; peipengbing1990@126.com

**Keywords:** upwelling, diluted water, ocean currents, Shantou Bay

## Abstract

The seasonal monsoon reversal drives runoff and current variability along the East Asian coast, intensifying eutrophication from terrestrial nutrients. However, phytoplankton responses to these combined pressures remain poorly understood. This study analyzed their effects using partial least-squares path modeling (PLS-PM) and generalized additive models (GAMs), based on 2021 data from Shantou Bay in the Taiwan Strait, a region with complex currents and significant nutrient inputs. A total of 359 phytoplankton species were identified, with seasonal mean abundances ranging from 6.76 × 10^6^ to 57.36 × 10^6^ cells m^−3^. Ocean currents and riverine runoff drive the seasonal turnover of dominant species by modulating the temperature and salinity. In summer, the exceptionally high phytoplankton abundance in the southwestern Taiwan Strait is driven by nutrient-rich terrestrial inputs, upwelling-induced thermal inhibition, and thermocline stratification from upwelling and offshore warm waters. The phytoplankton abundance and distribution were strongly correlated with the seasonal current and runoff-driven water masses. The PLS-PM results confirm that phytoplankton dynamics are regulated by currents and terrestrial nutrient inputs altering the hydrological and chemical environments, highlighting temperature and salinity as dominant controlling factors in eutrophic coastal zones.

## 1. Introduction

Phytoplankton form the foundation of marine food webs and play a central role in global biogeochemical cycles [1,2], particularly in coastal and estuarine ecosystems where environmental variability is pronounced [3]. Eutrophication driven by excessive land-based pollution has become a widespread global issue in estuarine waters [4,5,6]. Such nutrient enrichment often triggers phytoplankton blooms by stimulating primary productivity [4,6]. Crucially, land-based nutrient loading also elevates the original N/P ratio in marine environments, leading to a deviation from the Redfield ratio [7,8]. The resulting stoichiometric imbalance favors the dominance of small species with superior phosphorus adsorption capabilities, ultimately promoting a trend toward phytoplankton miniaturization [9,10,11]. Consequently, these anthropogenic shifts in stoichiometry and community size structure impose strong selective pressures, fundamentally altering the mechanisms of phytoplankton assemblage in coastal waters.

Open estuaries, situated at the interface between terrestrial and oceanic systems, are characterized by strong gradients in salinity, nutrients, turbidity, and hydrodynamics [3,12,13,14]. These gradients create highly dynamic habitats in which phytoplankton communities respond rapidly to both physical forcing and biogeochemical processes [15,16,17,18,19,20]. Unlike river-dominated or enclosed estuaries, open estuarine systems are strongly influenced by offshore water masses and large-scale ocean circulation [21,22]. The interaction between coastal currents, upwelling, and river discharge generates complex hydrological regimes that drive spatiotemporal variability in the environmental conditions [12,23,24]. Western boundary currents amplify the variablility through the interaction of warm saline waters with cooler, nutrient-rich coastal flows [25,26]. The pronounced density contrasts arising from this convergence drive the formation of stratification [25,26,27,28]. While thermocline stratification restricts the vertical transport of nutrients and reduces surface availability [28,29], runoff can reinforce this layering by inducing salinity gradients [30]. Stratification provides phytoplankton with stable light conditions, facilitating blooms when accompanied by a sufficient nutrient supply [31]. Thus, the intricate coupling of physical hydrodynamics and biogeochemical processes in open estuaries serves as a critical driver for phytoplankton dynamics and ecosystem productivity.

We hypothesize that seasonal hydrological regimes, mediated by currents and terrestrial runoff, regulate phytoplankton assemblies through temperature, salinity, and irradiance filtering, while nutrient enrichment amplifies bloom dynamics.

The southwestern Taiwan Strait (TWS) represents one of the most dynamic coastal systems in the Northwest Pacific. Here, the seasonal alternation and interaction of distinct water masses, including the Zhejiang–Fujian Coastal Water (ZFCW), South China Sea Warm Water (SCSWW), Kuroshio Branch Water (KBW), and Dongshan–Nan’ao Upwelling Water (DUW), create a heterogeneous environmental landscape [25,32,33,34,35,36,37,38]. This hydrodynamic complexity offers an ideal natural laboratory to examine how large-scale physical forcing modulates the distribution and coexistence of microbial taxa [25,39,40].

While the bottom-up controls of nutrients, light, and temperature in regulating phytoplankton dynamics are well-established [13,20,41,42,43,44,45,46], recent studies have emphasized that hydrodynamic processes are equally fundamental. They shape the community assembly by altering local environmental conditions and mediating material transport [47,48,49,50,51]. Consequently, the interaction between physical forcing and species-specific physiological traits is hypothesized to determine biodiversity patterns and the seasonal turnover of dominant taxa [51,52,53].

Shantou Bay (STB), located in the southwestern TWS, exemplifies these dynamics. As a partially open estuary, it is subjected to the dual pressure of severe eutrophication from terrestrial loading [54] and the intrusion of multiple oceanic water masses [37,40,55]. Despite its ecological significance, the specific mechanisms linking hydrological variability to phytoplankton assemblages in this region remain insufficiently understood.

In this study, we investigated the seasonal dynamics of phytoplankton communities in STB to test the hypothesis that hydrological shifts drive phytoplankton assemblages by altering the local environmental conditions and mediating material transport. By integrating field observations with statistical modeling, we aimed to characterize the seasonal hydrological and environmental variability, disentangle the effects of environmental filtering versus hydrodynamic transport on the species diversity and turnover, and elucidate the mechanisms linking hydrodynamics to the phytoplankton community structure. These findings will advance our understanding of biodiversity maintenance and ecosystem functioning in open estuarine systems influenced by complex hydrodynamics.

## 2. Methods

### 2.1. Sampling

**Figure 1:**
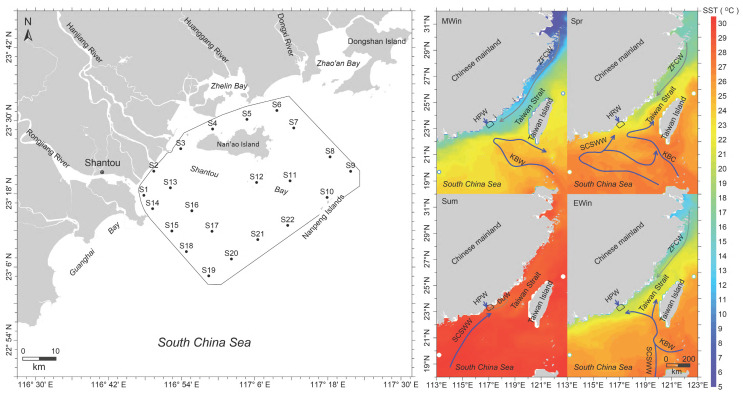
Map of the study area showing the sampling stations and the seasonal variations in the SST and surface circulation in the TWS (DUW, Dongshan–Nan’ao Upwelling Water; HPW, Hanjiang River Plume Water; KBW, Kuroshio Branch Water; ZFCW, Zhejiang–Fujian Coastal Water; SCSWW, South China Sea Warm Water) [26,36,40]. S1–S6, S13, and S14 represent the nearshore sites of the estuary; S8, S9 and S19–S22 are the offshore sites; while S7, S11, S12, and S15–S17 serve as the interactive sites.

Samplings were performed during four seasonal cruises in STB (23°4.2′–23°32.4′ N, 116°47.4′–1), with 22 sites distributed from the estuarine region to the waters around the Nanpeng Islands (Figure 1). The cruises were conducted during 13–15 January, 25–27 April, 1–3 August, and 6–9 December of 2021 on the RV “Haijian 304”.

A total of 88 water column samples were obtained in sterile 1 L dark bottles with Lugol’s. To assess the overall abundance of phytoplankton, discrete samples were collected at each designated depth (every 5 m) and three sub-samples from these depths were pooled to form a single composite sample (Figure S1). Following the Utermöhl settling technique, the water column samples were concentrated from 1 L to 30 mL through sedimentation for 48 h in the dark. Phytoplankton identification and enumeration were performed using an inverted microscope (Leica DMI3000B, Leica Microsystems, Wetzlar, Germany) at 200× magnification. A minimum of 400 cells per sample were counted to ensure statistical reliability. Taxonomic resolution was restricted to the morphospecies level based on Pacifica species guides [56,57] and AlgaeBase [58]. Simultaneously, vertical profiles of the hydrology, environment and phytoplankton were collected using CTD48M (SeaSun-Tech, Trappenkamp, Germany) from the seabed to the surface at 0.5 m intervals. The measured parameters included the temperature (Temp °C), salinity (Sal) and chlorophyll *a* (Chl *a*, mg m^−3^), while the turbidity (Turbid, FTU) and dissolved oxygen (DO, g m^−3^) were recorded specifically for the surface layer. The sampling and analytical procedures adhered to the national standards of the PRC for marine monitoring (GB 17378-2007). The parameters measured also included dissolved inorganic nitrogen (DIN, mmol m^−3^), and soluble reactive phosphate (SRP, mmol m^−3^) at the sea surface. The sea surface temperatures (SSTs) of the TWS were downloaded from China Ocean Real-Time Analysis, Northwest Pacific (CORTA 1.0-WNP, http://mds.nmdis.org.cn/ accessed on 1 January 2022), with daily resolution products. Surface current (SC) data for the TWS were obtained from China Ocean ReAnalysis (CORA v1.0, http://mds.nmdis.org.cn/ accessed on 1 January 2022) with monthly resolution products.

### 2.2. Data Analysis

The diversity (*H′*) and the dominant degree (*Y*) of phytoplankton were calculated using the following equations (Equations (1) and (2), respectively) [59,60]:

(1) $$H^{\prime }=-\sum_{i=1}^{S}\frac{n_{i}}{N}\log_{2}\frac{n_{i}}{N}$$

(2) $$Y=\frac{n_{i}}{N}\times f_{i}$$

In the equations above, *S* is the total species number; *n_i_* is the individual amount of species *i*; *N* is the total individual amount; and *f*_i_ is the occurrence frequency of the species in the samples. A species with a *Y* of more than 0.02 in a block is considered the block dominant species; a species with a *Y* of more than 0.02 in all blocks during a season is termed the seasonal dominant species [60]; and a species dominating the communities in all seasons is referred to as the annual dominant species.

To normalize skewed distributions, the phytoplankton abundance and normalized environmental data were log-transformed for use in subsequent analyses. The Spearman and Pearson correlations between the phytoplankton and environmental variables were analyzed in R 4.3.1 [61]. The structure of the phytoplankton community was represented by hierarchical clustering in R 4.3.1 [40]. A distance-based redundancy analysis (*db*-RDA) and Mental’s test, based on the Bray–Curtis’s similarity matrix, were applied to determine the environmental factors shaping the phytoplankton community structure in R 4.3.1 [40,62]. As species responses to environmental gradients are often complex, a generalized additive model (GAM) was introduced as a non-parametric approach using package mgcv 1.9.1 [63]. Unlike conventional regression techniques, the model is not restricted by specific functional relationships or underlying statistical distribution assumptions, allowing it to effectively capture the nonlinear response features of key species and groups’ abundances [64]. Finally, given the high collinearity and interactive effects among environmental variables, a partial least-squares path model (PLS-PM) was utilized to construct a multivariate network, further revealing the direct and indirect pathways through which environmental factors (monsoons, currents, hydrological conditions, land-based pollutions, nutrients) influence phytoplankton communities with the pacakage plspm 0.6.0 [65]. For visual representation, the seasonal distributions of SST, surface circulation, phytoplankton abundance, mass and ecological clusters were depicted using Surfer 14 [66].

## 3. Results

### 3.1. Species Composition

**Figure 2:**
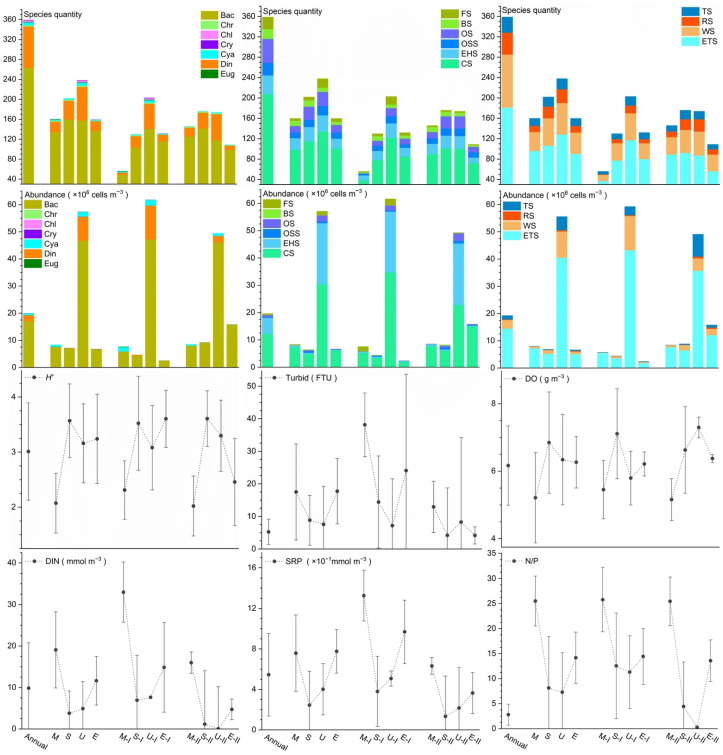
Seasonal and block-based means of phytoplankton abundance, species richness, diversity (*H’*), and surface environmental variables (M, min-winter; S, spring; U, summer; E, early winter; season-I/season-II, nearshore/offshore block in the season; Bac, Bacillariophyceae; Din, Dinophyta; Chr, Chrysophyceae; Cyn, Cyanobacteria; Chl, Chlorophyta; Cry, Cryptophyta; Eug, Euglenophyta; BS, brackish species; CS, coastal species; EHS, euryhaline species; FS, freshwater species; OS, oceanic species; OSS, open sea species; ETS, eurythermal species; TS, temperate species; RS, tropical species; WS, warm-water species).

A total of 359 species belonging to 128 genera were identified in Shantou Bay (STB), including 263 species from 89 genera of Bacillariophyceae, 83 species from 27 genera of Dinophyta, six species from six genera of Cyanobacteria, three species from three genera of Chlorophyta, two species from two genera of Chrysophyceae, one species from one genus of Cryptophyta, and one species from one genus of Euglenophyta (Table S1). Between spring and summer, the number of species ranged from 202 to 238 species, compared to only 160 species in winter (Figure 2). The species richness of Bacillariophyceae exhibited a similar seasonal pattern to that of the total phytoplankton, and the Dinophyta species in summer (68 species) were richer than in early winter to spring (20-38 species). The seasonal variation in the species number was more pronounced in nearshore communities than in offshore communities. Ecologically, phytoplankton are primarily composed of coastal species and eurythermal species, which account for more than half of the total species count, respectively. The distributions of tropical and oceanic species were higher in spring and summer (23–27 tropical species and 26–28 oceanic species) compared to winter (12–13 tropical species and 13–15 oceanic species). Freshwater species were more numerous in summer (18 species) compared to other seasons (5–7 species).

### 3.2. Community Structure and Environmental Conditions

**Figure 3:**
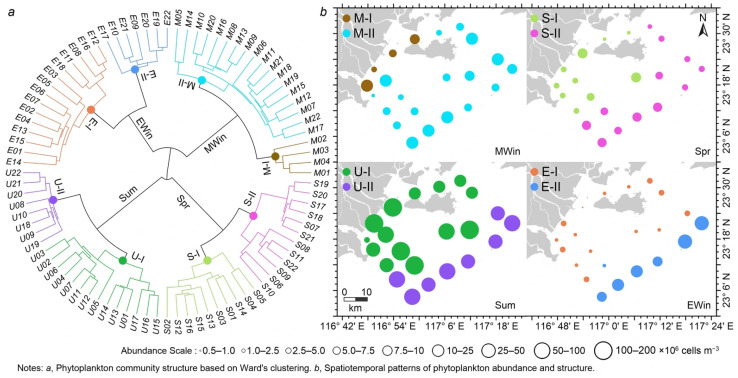
Seasonal variations in phytoplankton blocks and abundance (the abbreviations are shown Dataset S2).

Following Ward clustering, the phytoplankton communities showed significant differences across seasons and between nearshore and offshore locations in each season (Figure 3).

The total phytoplankton abundance was categorized into two seasonal clusters: the winter–spring cluster (mean values were 6.76–8.38 × 10^6^ cells m^−3^) and the summer cluster (57.36 × 10^6^ cells m^−3^) (Figure 2). Bacillariophyceae, Dinophyta, Cyanobacteria, and Chlorophyta exhibited a similar seasonal trend with the total phytoplankton in quantity. In terms of seasonal quantity composition, Bacillariophyceae, Dinophyta, and Cyanobacteria accounted for 81.4% to 99.4%, 0.5% to 15.2%, and 0.3% to 9.4% of the total phytoplankton, respectively.

Spatially, the offshore block exhibited a higher total phytoplankton abundance than the nearshore block during the same season from early winter to spring, while the trend was reversed in summer (Figure 2). The Bacillariophyceae abundance, ranging from 2.47 × 10^6^ cells m^−3^ to 46.09 × 10^6^ cells m^−3^, exhibited a similar spatial pattern with the total phytoplankton. The abundances of Dinophyta and Cyanobacteria in the nearshore block were more than those of the offshore block in each season. The Dinophyta abundance accounted for 20.3% of the total phytoplankton abundance in the nearshore block during summer. The Cyanobacteria abundance accounted for 21.6% of the total phytoplankton abundance in the nearshore block in min-winter. Additionally, the Bacillariophyceae abundance accounted for less than 80% of the total phytoplankton abundance in the nearshore block in min-winter and summer.

Ecologically, the seasonal abundances of phytoplankton groups based on their temperature or salinity tolerance exhibited a similar trend to the total phytoplankton (Figure 2). Coastal species and eurythermal species were the most abundant groups in terms of thermal tolerance and salinity tolerance, respectively. They accounted for 52.9% to 90.9% and 70.5% to 86.0% of the total phytoplankton abundance during each season. For euryhaline species blooming, the abundance of coastal species accounted for less than half of the total phytoplankton abundance in summer. The contribution to the total phytoplankton abundance for oceanic species was more than 5% in the offshore block during spring and summer. Freshwater species were more abundant in the nearshore blocks during min-winter (1.80 × 10^6^ cells m^−3^) and summer (2.38 × 10^6^ cells m^−3^) than in other blocks (0.003–0.29 × 10^6^ cells m^−3^). Temperature species accounted for more than 10% of the total phytoplankton abundance in the offshore block during summer. The abundance of warm-water species in the nearshore block (9.63 × 10^6^ cells m^−3^) was greater than that in the offshore block (4.54 × 10^6^ cells m^−3^) during summer, which was different from the other seasons.

The seasonal mean values of Shannon’s index ranged from 2.07 to 3.57, while Pilson’s index ranged from 0.40 to 0.62 (Figure 2). The spatial variations in the two indices were significant in early winter.

**Figure 4:**
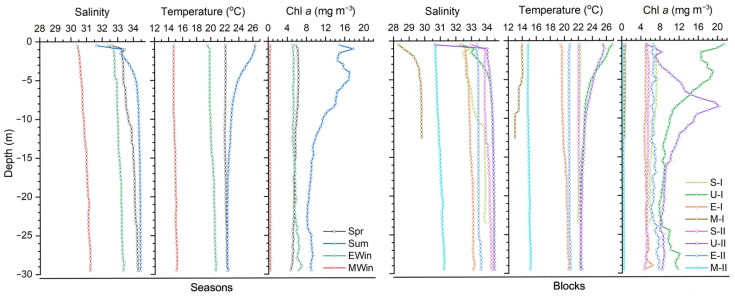
Mean vertical profiles of temperature, salinity and Chl *a* in STB.

The seasonal and spatial variations in the environmental variables are shown in Figure 2 and Figure 4. The sea surface Chl *a* (SSC), with seasonal means ranging from 0.43 mg m^−3^ to 14.89 mg m^−3^, was the highest in the nearshore block during summer (21.37 mg m^−3^), the lowest in min-winter (0.39–0.73 mg m^−3^), and similar in other blocks (5.33–8.25 mg m^−3^). The seasonal mean value of the sea surface salinity (SSS) in min-winter (30.42) was lower than in other seasons (31.64–32.39). The SSS in the nearshore block was also lower than in the offshore block during each season. The SST, with seasonal means ranging from 14.75 °C to 26.29 °C, was higher in the offshore block than in the nearshore block during winter; this trend reversed from spring to summer.

The mean vertical profiles of these three variables (Figure 4) revealed that the nearshore bay exhibited stratification tendencies driven by vertical salinity variations except for during early winter. During summer, the thermal stratification was pronounced. In the season, the vertical Chl *a* distribution showed marked variability, with concentrations exceeding >10 mg m^−3^ in the upper 10 m of the nearshore region and between 4 and 15.5 m in the offshore bay.

The turbidity, with seasonal means ranging from 7.55 FTU to 17.73 FTU, exceeded 10 FTU in the nearshore blocks during early winter to spring, and in the offshore block during min-winter. The seasonal mean values of DO ranged from 5.21 g m^−3^ to 6.85 g m^−3^. The DO levels in the nearshore block and the offshore block were similar during early winter (6.21–6.38 g m^−3^) and mid-winter (5.16–5.45 g m^−3^). In spring, the DO level was lower in the nearshore block, while the spatial trend was reversed in summer.

The pattern of DIN, SRP, and their ratio (N/P) exhibited similar temporal and spatial trends (Pearson correlation, *R* ≥ 0.69, *p* ≤ 0.01), and were inversely related to those of salinity (Pearson correlation, *R* ≤ −0.60, *p* ≤ 0.01). Their seasonal mean values in winter (DIN: 11.6–19.1 mmol m^−3^, SRP: 0.75–0.78 mmol m^−3^, N/P: 14.2–25.5) were higher than those in spring and summer (DIN: 3.80–4.88 mmol m^−3^, SRP: 0.24–0.40 mmol m^−3^, N/P: 7.3–8.1). The DIN in the nearshore block during min-winter was significantly higher than in the other blocks, and the values in the offshore blocks during spring and summer were lower. The SRP values in the nearshore blocks during the four seasons and the offshore block in min-winter were higher than those in the other three blocks. The N/P in the nearshore blocks during the four seasons and the offshore blocks in winter were higher than those in the other two blocks.

### 3.3. Dominant Species

**Figure 5:**
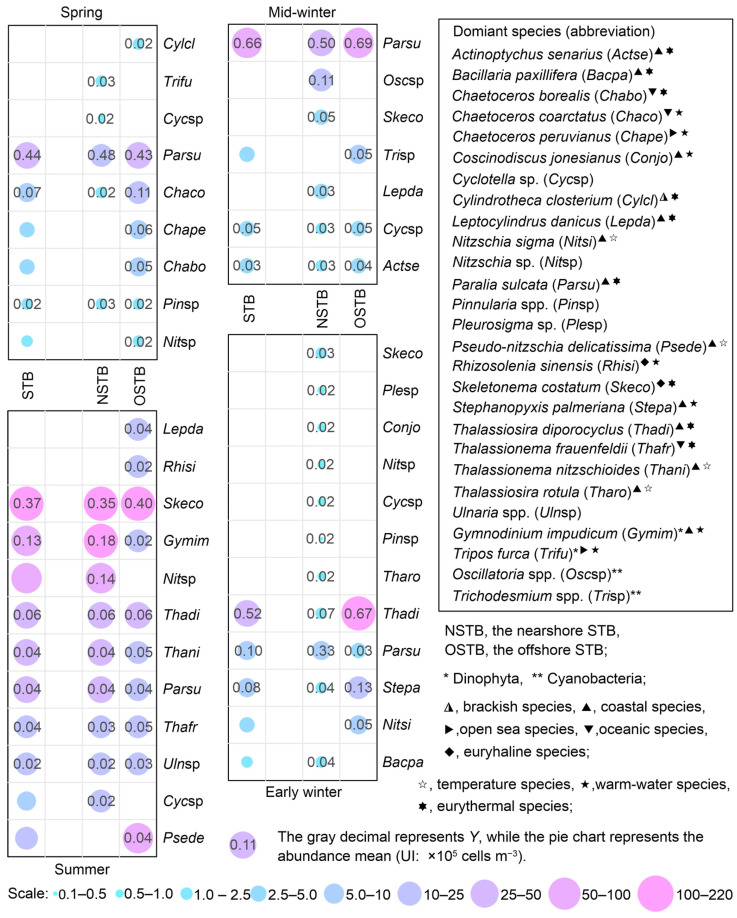
The patterns of abundance and degree of dominance for dominant species.

The phytoplankton abundance varied seasonally, with *Paralia sulcata* identified as the annual dominant species and eleven taxa as seasonal dominants, including *Cyclotella* sp. and *Actinoptychus senarius* in min-winter; *Chaetoceros coarctatus* and *Pinnularia* spp. in spring; *Skeletonema costatum*, *Gymnodinium impudicum*, *Thalassiosira diporocyclus*, *Thalassionema frauenfeldii*, *T. nitzschioides* and *Ulnaria* spp. in summer; and *Thalassiosira diporocyclus* and *Stephanopyxis palmeriana* in early winter (Figure 5). Based on the mean values, the total of all the dominant species comprised 55.4–74.0% of the total phytoplankton abundance. *P. sulcata* (seasonal mean: 7.0 × 10^5^–5.52 × 10^6^ cells m^−3^, block mean: 4.3 × 10^5^–5.90 × 10^6^ cells m^−3^) held more than 30% of the total phytoplankton abundance from min-winter to spring and in the nearshore block during early winter. *S. costatum* (seasonal mean: 4.0 × 10^4^–20.97 × 10^6^ cells m^−3^, block mean: 6.0 × 10^3^–21.54 × 10^6^ cells m^−3^) showed significant dominance in summer. *T. diporocyclus* (seasonal mean: 1.0 × 10^3^–3.50 × 10^6^ cells m^−3^, block mean: 0–10.63 × 10^6^ cells m^−3^) obviously aggregated with *Stephanopyxis palmeriana* together in the offshore block during early winter. *Chaetoceros coarctatus* exhibited a similar spatial pattern in spring. *G. impudicum* (block mean: 7.0 × 10^3^–11.8 × 10^6^ cells m^−3^) was just identified in summer and in the nearshore block during spring. The dominant species showed a bias toward being more abundant in the nearshore block. Additionally, some species were only dominant in the nearshore or offshore blocks. The species dominant in offshore blocks included *Chaetoceros borealis*, *C. peruvianus* and *Cylindrotheca closterium* in spring; *Leptocylindrus danicus*, Nitzschia sp., *Pseudonitzschia delicatissima* and *Rhizosolenia sinensis* in summer; *Nitzschia sigma* in early winter; and *Trichodesmium* spp. in min-winter. For nearshore blocks, the dominant species were *Tripos furca* in spring; *Nitzschia* sp. in summer; *Bacillaria paxillifera*, *Coscinodiscopsis jonesiana*, *Nitzschia* sp., *Pinnularia* spp., *Pleurosigma* sp. and *Thalassiosira rotula* in early winter; and *Oscillatoria* spp. in min-winter.

The predominance of eurythermal and coastal species (ES: eight species, CS: 12 species) indicated that STB has significant thermal variation and a relatively low salinity (Figure 5). Specifically, the dominance of temperature species in summer and early winter points to cold-water intrusions, while the abundant presence of oceanic species suggests substantial high-salinity water inputs driven by ocean currents.

### 3.4. Relationships Between Phytoplankton and Environmental Conditions

**Figure 6:**
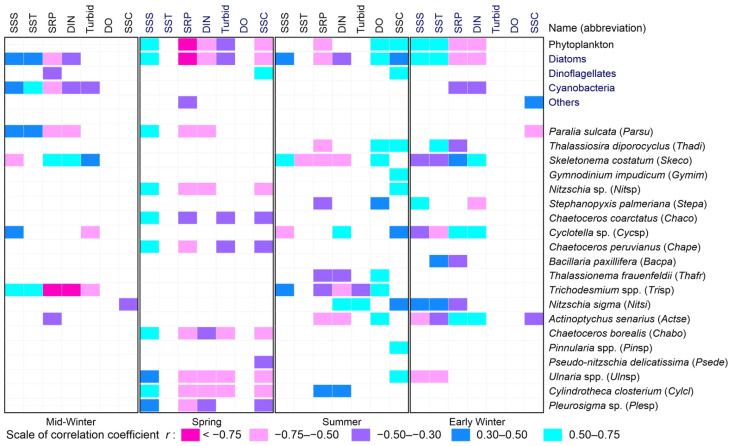
The relationship between phytoplankton abundance and the environmental variables of the sea surface (the abbreviations for dominant species and taxa groups are shown in Figure 2 and Figure 4; Phyto, phytoplankton).

The results of the Spearman correlation analysis indicated that the total phytoplankton abundance increased with salinity and decreased with nutrients in spring and early winter (Figure 6). The abundance trends of Bacillariophyceae across all seasons, as well as that of Cyanobacteria in winter, were similar to the total phytoplankton abundance. From min-winter to early winter, there were two, six, two, and one dominant species displaying similar relationships to the environmental variables. The correlation between the *Skeletonema costatum* abundance and the environmental variables was positive in summer, but reversed in winter, reflecting seasonal physiological and hydrodynamic influences. The species abundance decreased with temperature from summer through early winter. *Thalassiosira diporocyclus* exhibited a significant negative correlation with the SRP in summer and early winter. *Gymnodinium impudicum* was correlated only with Chl *a* in summer. *Cyclotella* sp., *Nitzschia sigma* and *Ulnaria* spp. favored a high N/P.

**Figure 7:**
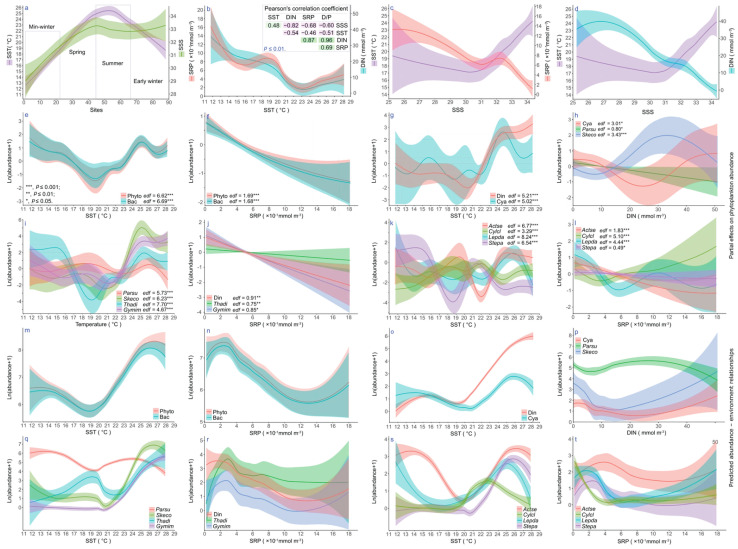
Interrelationships among environmental variables (**a**–**d**), partial effects of environmental variables on phytoplankton abundances (**e**–**l**) and bivariate relationships between phytoplankton abundance and environmental variables (**m**–**t**); predictor importance evaluated based on estimated degrees of freedom (*edf*) and *p*-values.

The results of the GAM analysis indicate that the temperature and nutrients significantly influence the phytoplankton abundance in STB (Table 1; Figure 7e–l). The total phytoplankton abundance increased with warming between 19.5 °C and 25 °C, and the Bacillariophyceae abundance showed a similar pattern. The Dinophyta abundance rose with increasing temperatures above 22 °C. In contrast, the Cyanobacteria abundance exhibited a unimodal response to temperature, first decreasing and then increasing between 22 °C and 24.5 °C. Seasonal variations in the abundances of dominant species closely tracked the temperature fluctuations.

The *Paralia sulcata* abundance began to increase at 13.8 °C, declined above 18.4 °C, rose again above 22.8 °C, and decreased once more above 26 °C. *Skeletonema costatum*, *Gymnodinium impudicum*, and *Thalassiosira diporocyclus* all showed positive responses to warming within the ranges of 20.8–25.5 °C, 21.1–25 °C, and 19.1–25 °C, respectively. *Actinoptychus senarius* exhibited two favorable temperature windows: 15.8–19.4 °C and 22–25 °C. In contrast, *Leptocylindrus danicus* was negatively affected by temperatures below 14.3 °C and above 25.9 °C. The *Cylindrotheca closterium* abundance decreased in two temperature intervals (18.1–20.3 °C and 22–24.5 °C), as did *Stephanopyxis palmeriana* (15.8–19.4 °C and 22–25 °C). These patterns confirm that temperature is the primary driver of the phytoplankton abundance dynamics in STB.

Nutrients also play an important role in shaping the phytoplankton community. The total phytoplankton abundance decreased with an increasing SRP concentration. Similar negative trends were observed for Bacillariophyceae, Dinophyta, and four dominant species, including *T. diporocyclus*, *G. impudicum*, *A. senarius*, and *S. palmeriana*. *L. danicus* and *C. Closterium* also declined with rising SRP, but only below thresholds of 0.55 mmol m^−3^ and 0.34 mmol m^−3^, respectively. The DIN also influenced the community composition. The *P. sulcata* abundance decreased with increasing DIN. Cyanobacteria declined at DIN concentrations below 24.2 mmol m^−3^. In contrast, *S. costatum* thrived within a DIN range of 7.7–32.7 mmol m^−3^. Furthermore, the mismatch between the optimal SSTs of dominant species and high nutrients, due to negative relationships between hydrological factors and nutrients, acted as a barrier against those bloom outbreaks.

A comparison between the partial effects of environmental variables (Figure 7e–l) and bivariate relationships (Figure 7q–t) revealed that the temperature at which the predicated phytoplankton abundance peaked was 0.5–2 °C higher than the optimal temperature for all functional groups and most dominant species. Bivariate analyses further showed that the DIN exerted top-down control on Cyanobacteria at concentrations below 22.4 mmol m^−3^, on *P. sulcata* below 4.5 mmol m^−3^, and on *S. costatum* below 16.5 mmol m^−3^. The *C. closterium* abundance decreased with increasing SRP below 4 mmol m^−3^, whereas other functional groups and dominant species responded positively to low SRP concentrations (typically <0.15–0.40 mmol m^−3^). These results reveal that the interplay between hydrological conditions and nutrient availability caused the temperature for the peak abundance of dominant species to deviate from their optimal values. Moreover, the relatively wide optimal temperature range and low nitrogen-limiting concentration of *S. costatum* make the mismatch between its optimal SST and a high DIN highly vulnerable to disruption. Coupled with the environmental interactions, this renders the species highly susceptible to blooms in the eutrophic STB (Figure 7).

**Figure 8:**
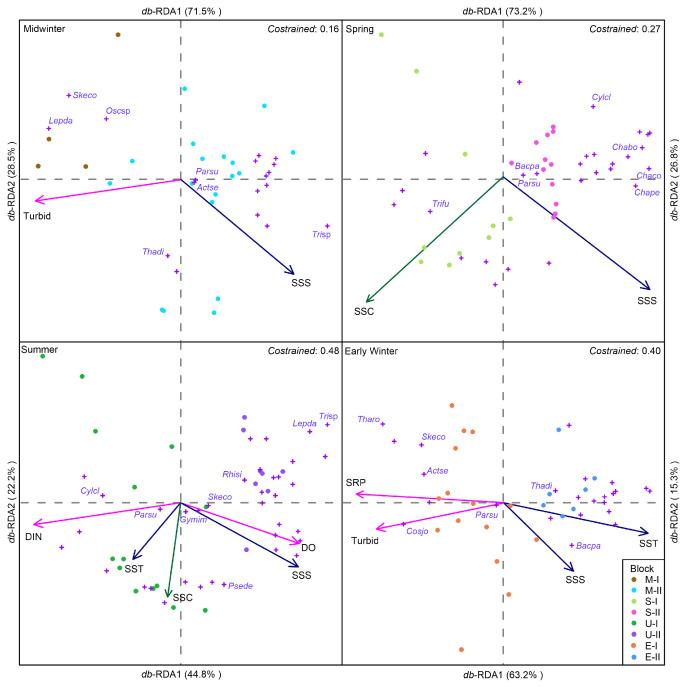
*db*-RDA analysis result of seasonal phytoplankton communities and sea surface environmental impactors (the abbreviations for dominant species are shown in Figure 4).

The results of *db*-RDA exhibited that the phytoplankton communities were closely related to the environment factors, explaining 16% to 47% of the variation in these communities (Figure 8 and Figure S2). The spatial patterns of phytoplankton communities were consistently influenced by salinity. The effects of temperature and nutrients were more pronounced in summer and early winter, while the influence of turbidity was more pronounced in winter.

The results of *db*-RDA also indicated the spatial patterns of the dominant species in relation to environmental impactors. A relatively high salinity restricted nearshore dominant species from assembling in the offshore waters and allowed offshore dominant species to thrive in that zone. However, the spatial distributions of seasonal dominant species showed weak relationships with environmental factors. Nevertheless, nearshore dominant species such as *Bacillaria paxillifera* and seasonal dominant species like *Stephanopyxis palmeriana* and *Thalassiosira diporocyclus* exhibited a preference for a relatively high salinity in early winter. *Skeletonema costatum*, *Actinoptychus senarius* and *Thalassiosira rotula* exhibited negative relationships with temperature during the season. In summer, *Leptocylindrus danicus* and *Trichodesmium* spp. exhibited a bias toward relatively cold waters.

**Figure 9:**
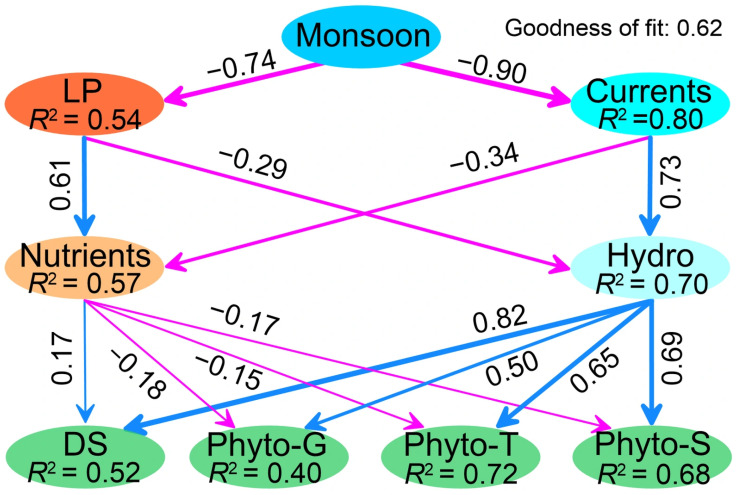
Path diagrams for annual PLS-PMs of phytoplankton ecosystem (currents embodying ZFCW, SCSWW, and DUW; LP, land-based pollutions containing city discharge and inputs of total nitrogen and phosphorus from Hangjiang River and Rongjiang River; nutrients containing DIN and SRP; Hydro, hydrological variables, including SST and SSS; DS, dominant species abundance; Phyto-G, Phyto-T and Phyto-S, abundances of phytoplankton groups based on taxa, temperature tolerance and salinity tolerance. The data used for the PLS-PMs are presented in Dataset S3).

The PLS-PM results indicated that the seasonal variations in land-based pollutant inputs and ocean currents, influenced by the monsoon reversals, regulated the spatial and temporal patterns of nutrients and hydrological conditions, which dominated the seasonal and spatial structure of phytoplankton communities (Figure 9). The nutrient concentrations were primarily influenced by land-based pollutant inputs, while the hydrological conditions were mainly dominated by seasonal shifts in the ocean currents. The hydrological conditions significantly impacted the structure of the phytoplankton communities, leading to temporal and spatial differences in their ecological adaptations. There were weak negative correlations between high nutrient concentrations and the phytoplankton communities, but a positive correlation with dominant species, indicating that high nutrient concentrations favor the dominance of these species.

## 4. Discussion

### 4.1. Seasonal Reversals of Currents and Runoff Drive Hydrological and Environmental Regimes

Our findings reveal an inverse relationship between temperature/salinity and nutrient/suspended matter concentrations in STB, driven by the seasonal alternation between coastal and warm currents. In winter, the low salinity and temperature, coupled with a high turbidity and nutrients in STB, suggest ZFCW invasion [32,33,40]. During spring and summer, the reduction in the turbidity and nutrients coinciding with warming and salinization in the bay aligns with the intrusion of warm-water masses [25,34,35]. The mean salinity greater than 34 in the offshore zone is consistent with Kuroshio water intrusion into the southwestern TWS in spring [36] and the upwelling event during summer 2021, which was characterized by an SST approximately 1 °C lower than that in the same season of 2022 [35,37,55,67]. Moreover, the consistently higher concentrations of nutrients and suspended matter in the nearshore bay compared to the offshore zone across all seasons highlight that local terrestrial input is a key factor driving eutrophication in the bay [68]. Nearshore salinity stratification indicates the influence of river runoff on the STB hydrographic structure [27]. Furthermore, the thermocline stratification resulting from surface wind-wave mixing between warm surface waters and upwelled waters inhibits the upwelling from penetrating the thermocline to reach the surface [27,28]. This is consistent with the SCSWW invasion during summer, facilitating the formation of the coastal upwelling front in the southwest TWS [69]. The seasonal alternation of currents governs the overall hydrological and environmental variability in the bay, while the local terrestrial input imparts distinct characteristics to the nearshore zone, resulting in perennial eutrophication and high suspended matter loads.

### 4.2. Species Richness Responds to Complex Hydrodynamics

STB emerges as a hotspot of phytoplankton diversity in Southeast China, with 359 recorded species. This richness exceeds that observed in semi-enclosed bays of the region, while its seasonal assemblages, ranging from 160 to 238 species, mirror the patterns found in the Taiwan Strait (Table S2). Hwang [39] suggests that the high plankton diversity of the TWS can be attributed to the year-round presence of local species and the aggregation of taxa from subtropical, tropical, and temperate water masses, a process facilitated as monsoon-driven currents and the Kuroshio Branch Current appear to mask the effect of river discharge in the region. This pattern was confirmed in STB, where tropical, temperate, and oceanic species each contribute over 7.5% to the seasonal phytoplankton assemblages, in the context of a community dominated by eurythermal coastal species and the seasonal replacement of the ZFCW, SCSWW, DUW, and KBW (Figure 1). The diverse hydrological and environmental gradients generated by the year-round interaction of distinct water masses in the bay [70,71] provide more possibilities for the specialization of ecological traits [72], thereby fostering an exceptionally rich phytoplankton diversity. Moreover, the positive effect of oceanic currents on the diversity of rare phytoplankton [73], coupled with temperature as the most important driver of phytoplankton richness [20,74], helps to explain the high species richness in STB during spring and summer, which coincides with the intrusion of warm oceanic waters. Collectively, these findings indicate that the complex and unique hydrodynamic regime endows the southern coastal waters of the TWS with exceptionally rich phytoplankton species and a distinct seasonal trend in diversity.

### 4.3. Thermal Windows Drive Dominant Species Turnover

According to the Spearman correlation analysis, GAMs and PLS-PM analyses, the seasonal patterns of dominant species are driven by nutrients and hydrological conditions in STB. Temperature is a key driver for the seasonal species turnover in the bay. In STB, the dominant species exhibited three optimal SST windows: >15 °C, 18–22 °C and 25–26 °C, corresponding to mid-winter, spring/early winter, and summer, respectively. *Paralia sulcata* and *Actinoptychus senarius* displayed the three optimal SST ranges in the bay. The realized niche optimum for the former is defined as a sea temperature of 6.25 ± 3.50 °C and a salinity of 32.8 ± 0.52, based on spring data from the Yellow Sea [75]. For the latter, an optimal temperature of 14.8 °C has been reported off North Portugal [76].

The summer and early-winter dominant species, *Thalassiosira diporocyclus*, showed two potential optimal SSTs (15.0 °C and 25.3 °C), with distinct abundance peaks observed at 19.3 °C and >29.0 °C in STB. While this species grows at 15–27 °C in culture, field observations in Minamata Bay, Japan, restricted high cell densities to a narrow range of 21.0–21.2 °C and a salinity of 33.0–33.1 [77]. The species demonstrated significant presence in the ZFCW zone of the Taiwan Strait during the winter of 2016 [25]. Blooms were also recorded at the Taiwan Bank in early spring 2016 at temperatures of 15.95 ± 0.97 °C and salinities of 32.84 ± 0.77 [78], as well as temperatures ranging from 18.3 °C to 22.1 °C in Zhelin Bay (an inner bay northeast of STB) during winter [79]. Furthermore, *Stephanopyxis palmeriana*, dominant in early winter, also had an optimal SST in the low-temperature zone (>15 °C) in STB. This species is reported to prefer habitats below the pycnocline and exhibits a tolerance to low light intensities in stratified waters around Japan [80]. These findings suggest that these four species prefer cold temperatures, and their abnormally high abundance often indicates cold-water intrusion in STB.

The summer dominant species, *Skeletonema costatum* and *Gymnodinium impudicum*, exhibited an optimal SST of 25 °C in STB. *S. costatum* was most abundant in STB at an SST of 26.4 °C and in most aquaculture zones of Fujian Province at SSTs ranging from 24.7 °C to 27.8 °C [81]. *G. impudicum* typically blooms at temperatures between 22 °C and 31 °C in eutrophic coastal waters [82], and its optimal SST has been confirmed in culture experiments [83]. The dominance of these summer species indicates that their success arises from the alignment of environmental temperatures with their physiological thermal optima.

However, *P. sulcata*, *A. senarius*, and *T. diporocyclus* also exhibited an optimal SST in the high-temperature zone (25–26 °C), which appears to contradict their preference for cold waters. During summer, the mean temperatures in the nearshore and offshore water columns characterized by high Chl *a* levels ranged between 23.2 °C and 24.2 °C (Figure 4). The optimal temperature range for the growth and metabolism of temperate diatom species is typically 20–24 °C [84]. Furthermore, enriched nutrients and sufficient irradiance often reduce phytoplankton temperature sensitivities [41,44,46,85]. The summer DIN and SRP levels in STB (means of 4.9 mmol m^−3^ and 0.24 mmol m^−3^, respectively; Figure 1) both exceeded the half-saturation constants for phytoplankton uptake (2.0 mmol m^−3^ for DIN and 0.20 mmol m^−3^ for SRP) [86]. The summer turbidity levels were the lowest in STB (Figure 2). These factors suggest that the abnormal abundance of these three species in summer resulted from the coupling of their inherent physiological patterns with upwelling-induced environmental conditions in this eutrophication-prone estuary.

These findings highlight that the turnover of dominant species is mediated by seasonal hydrological and environmental shifts, particularly temperature, that alter the physiological patterns of phytoplankton.

### 4.4. Dominant Species Turnover Responds to Hydrographic Variability

Hydrographic processes play a pivotal role in determining phytoplankton assemblages [47,87,88,89]. Tilstone et al. [87] reported that upwelling events cause the export of *Thalassiosira* spp. and *Skeletonema costatum* standing stocks from upwelling zones toward adjacent shelves in the Ría de Vigo, Spain. A similar phenomenon, characterized by the abnormal abundance of *S. costatum* and *Thalassiosira diporocyclus* during the 2021 upwelling period, was observed in Shantou Bay (Figure 4 and Figure 5). In winter, STB experiences higher turbidity and nutrient levels, accompanied by a lower temperature and salinity, due to the intrusion of the vertically well-mixed ZFCW (Figure 4) [40,68]. *P. sulcata* and *A. senarius* have been reported as dominant species in the surface sediments of the Taiwan Strait [90]. The high abundance of *P. sulcata* and *A. senarius* also indicates that the water column is vertically well-mixed in the STB, for the ZFCW intrusion during winter. Furthermore, it suggests that a high turbidity confers a competitive advantage to low-light-adapted benthic phytoplankton species. Therefore, the shift in dominant phytoplankton species reflects the coupled nature of oceanic physical and biogeochemical processes.

### 4.5. Phytoplankton Communities in Response to Hydrological Regime Shifts

The seasonal succession of phytoplankton communities in coastal ecosystems is rarely driven by single factors, but rather by the complex interplay of hydrological regimes and their resultant physicochemical gradients. In STB, the observed patterns can be mechanistically explained through the lens of environmental filtering and niche differentiation imposed by the alternating dominance of ZFCW, SCSWW, DNUW and HPW.

The proliferation of cold-water-preferring species confirms the cold-water invasion. Coastal and eurythermal species became more abundant than other functional groups in STB during winter (Figure 2). Specifically, coastal taxa favoring cool water, including *Thalassiosira diporocyclus*, *Stephanopuxis palmeriana* and *Paralia sulcata*, proliferated. Temperature and salinity are often regarded as the primary drivers of phytoplankton species replacement [13,91,92,93,94]. These observations indicate that ZFCW intrusion reshapes the local bay environment. In mid-winter, the intrusion of the ZFCW intensified, causing bay-wide temperatures to plummet to annual minima and the turbidity to surge to yearly peaks [32,33,40]. Concurrently, the benthic diatoms *P. sulcata* and *Actinoptychus senarius* maintained their dominance across the entire bay [90]. This highlights the ZFCW influence in the bay and indicates that the light limitation also governs the selection of phytoplankton species in high-turbidity coastal waters [43,45,48,49,90,95]. This also creates a mismatch pattern between high nutrients and a poor abundance of phytoplankton in winter. During summer, the annual maximum relative abundance of temperate species in offshore waters during the season confirms the injection of cool water by the upwelling warming [40,55]. The thermal optimum of *Leptocylindrus danicus* is generally suggested to be between 15 and 20 °C [96], and this species is observed to be exceptionally abundant when the temperature ranges from 9.9 °C to 13.8 °C in STB and San Jorge Gulf, Argentina [97]. The species aggregations with *P. sulcata* and *Thalassiosira diporocyclus* together in the season further confirmed the summer upwelling event. Moreover, *Cylindrotheca closterium* mainly assembled in the offshore STB with an abundance peak at an SST of 22.5 °C. The species is reported as a eutrophic benthic diatom with a thermal optimum at 20 °C [98], and its abundant presence in spring indicates the lingering effects of the ZFCW [34].

The presence of saline, warm-water-preferring species confirms the warm-water invasion. The STB environment during spring is characterized by rising temperatures and salinity coupled with nutrient drawdown associated with the SCSWW intrusion [34]. The increased presence of tropical and oceanic species compared to winter indicates the invasion of saline warm water, highlighted by oceanic/open-sea dominant species such as *Chaetoceros borealis*, *C. coarctatus*, *C. peruvianus*, and *Tripos furca* [52,99]. *C. peruvianus* along with three species dominant in summer (*Pseudonitzchia delicatissima*, *Thalassionema nitzschioides* and *T. frauenfeldii*) are abundant in the Kuroshio Extension and adjacent waters (2.11–22.95 °C, 33.17–34.77) [52]. *T. furca* can thrive across a wide temperature range (18–28 °C) [100], with the optimal salinity and temperature reported at 33.1–33.8 and 26.5–28.6 °C in the southern South China Sea [101]. Additionally, the *Trichodesmium* abundance was positively correlated with temperature, having optimal growth temperatures ranging from 25 °C to 30 °C [102]. The genus was reported as the indicator of Kuroshio intrusion [25,52]. Its aggregation in the offshore zone during mid-winter suggests that the invading ZFCW had mixed with warm waters [36,103]. Furthermore, *Rhizosolenia sinensis*, a species typically associated with Kuroshio waters [25], co-occurred with the genus in offshore waters during summer, further suggesting the influence of the SCSWW in the bay [25,40,52].

Terrestrial inputs also play a significant role in shaping the phytoplankton community. Both the diversity and abundance of freshwater species in the nearshore region reached their annual peak in summer, coinciding with the Hanjiang River discharge, which is more than double that of other seasons [104]. *Skeletonema costatum*, a summer dominant species, showed a strong tendency to the nearshore STB (Figure 4). It is characterized as a species thriving in eutrophic and relatively low-salinity environments [49,105,106,107,108]. Meanwhile, *Pseudo-nitzchia delicatissima*, *Thalassionema nitzschioides* and *T. frauenfeldii* were abundant in the bay during summer. The three species demonstrate a high tolerance for environmental fluctuations, maintaining numerical dominance across habitats ranging from tropical estuaries to the confluence of the Kuroshio and Oyashio currents [52,109,110,111,112]. Therefore, the highest salinity observed in the nearshore zone during this period suggests that the nearshore waters were well-mixed [69]. These findings indicate that these four species have a propensity to establish dominance under conditions of significant environmental variation.

In summary, the seasonal dynamics of the phytoplankton community in STB reflect species-level ecological filtering, matching physiological traits to the specific thermal, nutritional, and hydrodynamic regimes driven by water mass movements.

### 4.6. Hydrological and Environmental Drivers of Seasonal Phytoplankton Anomalies

During summer, the bay exhibited a phytoplankton bloom coinciding with the peak temperature and thermal stratification, most notably in nearshore waters (Figure 2 and Figure 4). Stratification facilitates phytoplankton blooms by enhancing their exposure to irradiance [12,31]. While warming generally promotes the growth rate of entire phytoplankton communities under different nutrient conditions [113,114], the positive effect of warming is significantly attenuated under in situ nutrient-limited conditions compared to nutrient-replete environments [42,44,114]. These factors collectively explain that the peak phytoplankton abundance observed in summer is the combined effect of favorable temperatures, continuous nutrient inputs, and stratification ensuring sufficient light availability.

Specific hydrological and environmental conditions drove *S. costatum* blooms in STB during the nitrogen-limited period. This indicates that a mismatch between optimal hydrological conditions and rich nutrients impedes the explosive blooming of this species [115]. This differs from the blooms in most of other Chinese bays occurring under a high N/P [10,116,117]. It also highlights the importance of controlling the nitrogen inputs to STB, particularly given that this ecological balance could be disrupted by warming, potentially inducing massive blooms [74,118]. Furthermore, *S. costatum* blooms can restrict the carbon uptake efficiency of phytoplanktivorous copepods [119]. Since zooplankton serve as essential food resources for many pelagic larval fishes [120], these dynamics suggest that warming exacerbates food shortages for higher trophic levels. Therefore, implementing stringent measures to mitigate terrestrial nutrients is a paramount ecological imperative.

Although upwelling injects nutrient-rich water to the bay [40], nutrients were depleted in the offshore zone (Figure 2). A similar pattern of a high biomass and depleted nutrients in upwelling zones has been reported in the southern TWS and the California Current System [51,53]. Hu et al. [51] suggest that this pattern is shaped by the rapid phytoplankton growth under favorable conditions (e.g., a column water temperature <25 °C), which leads to the consumption of most nutrients in the upwelled water. Consequently, the offshore STB showed a distinct seasonal shift toward dinoflagellates in summer, driven by a low N/P (Figure 2) and silicate levels [40], similar to the nitrate-driven dominance observed in the Benguela Upwelling System [121]. Furthermore, these indicate that estuaries transition from phosphorus- to nitrogen-limited states in upwelling regions, exhibiting distinct N:P ratios compared to other estuaries, and reinforces that upwelling estuaries are inherently more susceptible to harmful algal blooms due to their unique hydrological and environmental patterns.

Under sufficient light and favorable temperatures, *Gymnodinium impudicum* blooms in summer and tends to aggregate more in nearshore waters with high terrestrial dissolved organic matter (DOM) [122]. During the bloom, the species abundance reached 3.39 × 10^9^ cells m^−3^ after 23 days [83]. These findings suggest a transition from a senescing *S. costatum* bloom to a *G. impudicum* bloom. The preceding diatom bloom depleted the DIN, creating nitrogen-limiting conditions [123]. In this environment, abundant terrestrial DOM facilitated the subsequent dinoflagellate outbreak [122,123]. Ultimately, the occurrence of *G. impudicum* blooms is driven by the interaction of species competition, physiological traits, hydrological conditions, and specific nutrient environments, underscoring the critical need for the stringent mitigation of terrestrial nutrient inputs to prevent such ecological shifts.

Beyond the taxonomic shift toward dinoflagellates, excessive terrestrial nutrient inputs also drive phytoplankton miniaturization. For instance, the small dominant species *Cyclotella* sp., *Nitzschia sigma* and *Ularia* spp. favored high-N/P environments. These tended to dominate under phosphorus-limited conditions when the N/P ratio is elevated, for they possessing substantial surface-adsorbed phosphorus pools [9,10,11]. Additionally, the offshore dominant species *Pseudo-nitzschia* is less silicified than the *Chaetoceros* and *Thalassiosira* genera [53,124]. A taxonomic shift from siliceous to non-siliceous phytoplankton and/or lightly silicified diatoms due to nutrient limitation may be another contributing factor [53,121]. In summary, the seasonal phytoplankton dynamics in STB reflect the complex interplay of physiological traits, salinity, light, nutrients, and nutrient stoichiometry.

## 5. Conclusions

This study elucidates the spatial and temporal dynamics of phytoplankton assemblages in STB, emphasizing the biophysical coupling between complex hydrodynamics and terrestrial nutrient loading. Our findings demonstrate that community dynamics are intrinsically linked to the water mass variability within the southwestern TWS. Specifically, the seasonal alternation between riverine discharge and currents acts as a hydrodynamic filter, regulating phytoplankton succession by modulating the spatiotemporal regimes of temperature and salinity. The ZFCW intrusion introduces cold nutrient-rich water to select cold-affinity taxa, including *Thalassiosira diporocyclus*, *Stephanopyxis palmeriana*, *Paralia sulcata*, and *Actinoptychus senarius*. In contrast, the summer synergy of HPW, DUW, and SCSWW establishes a physically stratified regime, with an initially high nutrient availability that is rapidly depleted by diatom uptake, ultimately driving the system toward nitrogen limitation. Crucially, we identified a distinct two-stage bloom mechanism driven by upwelling in eutrophic estuaries. Initially, stratification driven by the interplay between upwelling and diluted water triggers rapid diatom proliferation, which acts as a “biological pump”, swiftly depleting the DIN and driving the system toward nitrogen limitation. This stoichiometric imbalance, coupled with DOM-enriched nearshore waters, creates a specific ecological niche that favors the subsequent succession to dinoflagellates. Furthermore, chronic terrestrial nutrient loading drives phytoplankton miniaturization, where environmental instability favors widespread species and opportunistic micro-species over larger taxa. Notably, the contrasting seasonal responses of dominant taxa highlight that species-specific physiological traits interact with hydrodynamic forcing to determine community structure (e.g., the reversed thermal correlation observed in *S. costatum*). Collectively, these results validate the hypothesis that seasonal hydrological shifts, mediated by currents and runoff, regulate phytoplankton diversity through temperature, salinity and irradiance filtering. Furthermore, nutrient enrichment amplifies bloom dynamics, and nutrient stoichiometry drives functional group succession. As warming disrupts historical nutrient and hydrological regimes, implementing stringent pollution management in upwelling estuaries is a paramount ecological imperative.

## Figures and Tables

**Table 1:** Statistical summary of generalized additive models (*AIC* denotes the Akaike information criterion; *GCV* denotes generalized cross-validation; the deviance of the weight-specific ingestion rate explained by the predictors involved is indicated by *DE*).

Species/Group	Model	*DE*	*R*	*GCV*	*AIC*
P hytoplankton	Ln(abundance + 1) ~ Season + s(SST) + s(SRP)	77.3%	0.86	0.46	172
Bacillariophyceae		75.4%	0.85	0.46	172
Dinophyta		80.0%	0.88	1.13	254
*Thalassiosira diporocyclus*		85.6%	0.92	1.21	258
*Gymnodinium impudicum*		81.7%	0.90	1.21	262
*Actinoptychus senarius*		78.6%	0.87	0.7	209
*Cylindrotheca closterium*		61.6%	0.75	0.62	199
*Leptocylindrus danicus*		63.3%	0.75	1.51	271
*Stephanopyxis palmeriana*		7 8.3%	0.87	0.83	226
Cyanobacteria	Ln(abundance + 1) ~ Season + s(SST) + s(DIN)	42.4%	0.60	3.15	342
*Paralia sulcata*		50.2%	0.67	1.09	251
*Skeletonema costatum*		86.1%	0.92	1.88	294

## Data Availability

The original data presented in the study are openly available in Zenodo. The dataset used for the sea surface temperatures and currents in the Taiwan Strait (Dataset S1).

## Supplementary Materials

The following supporting information can be downloaded at: https://www.mdpi.com/article/10.3390/microorganisms14061363/s1, Figure S1: The seasonal phytoplankton abundance in water column and layers; Figure S2: The relationships between seasonal phytoplankton communities and sea surface environmental impactors based on Mantel’s test; Dataset S1: The dataset used for the sea surface temperatures and currents in the Taiwan Strait; Dataset S2: The datasets used for the list of abbreviations; Dataset S3: the distribution of monsoons, water masses, runoff, land-based pollution inputs, environmental factors, and phytoplankton abundance; Table S1: the list of identified phytoplankton species from Shantou Bay in 2021; Table S2: and the marine phytoplankton species of Southeast China.

## Abbreviations

The following abbreviations are used in this manuscript:

TWS	Taiwan Strait
STB	Shantou Bay
DUW	Dongshan–Nan’ao Upwelling Water
HPW	Hanjiang River Plume Water
KBW	Kuroshio Branch Water
SCSWW	South China Sea Warm Water
ZFCW	Zhejiang–Fujian Coastal Water
SST	sea surface temperature
SSS	sea surface salinity
SSC	sea surface chlorophyll *a*
DIN	dissolved inorganic nitrogen
DO	sea surface dissolved oxygen
SRP	soluble reactive phosphate
Temp	temperature
Sal	salinity
Turbid	sea surface turbidity
Chl a	chlorophyll *a*
N/P	DIN/SRP ratio
*db* -RDA	distance-based redundancy analysis
GAM	generalized additive model
PLS-PM	partial least-squares path model
Chl	Chlorophyta
Chr	Chrysophyceae
Cry	Cryptophyta
Cya	Cyanobacteria
Bac	Bacillariophyceae
Din	Dinophyta
Eug	Euglenophyta
BS	brackish species
CS	coastal species
EHS	euryhaline species
FS	freshwater species
OS	oceanic species
OSS	open-sea species
ETS	eurythermal species
RS	tropical species
TS	temperate species
WS	warm-water species
*Actse*	*Actinoptychus senarius*
*Bacpa*	*Bacillaria paxillifera*
*Chabo*	*Chaetoceros borealis*
*Chaco*	*Chaetoceros coarctatus*
*Chape*	*Chaetoceros peruvianus*
*Cosjo*	*Coscinodiscus jonesianus*
*Cyc*sp	*Cyclotella* sp.
*Cylcl*	*Cylindrotheca closterium*
*Gymim*	*Gymnodinium impudicum*
*Lepda*	*Leptocylindrus danicus*
*Nitsi*	*Nitzschia sigma*
*Nit*sp	*Nitzschia* sp.
*Osc*sp	*Oscillatoria* spp.
*Parsu*	*Paralia sulcata*
*Pin*sp	*Pinnularia* spp.
*Ple*sp	*Pleurosigma* sp.
*Psede*	*Pseudo-nitzschia delicatissima*
*Rhisi*	*Rhizosolenia sinensis*
*Skeco*	*Skeletonema costatum*
*Stepa*	*Stephanopyxis palmeriana*
*Thafr*	*Thalassionema frauenfeldii*
*Thani*	*Thalassionema nitzschioides*
*Thadi*	*Thalassiosira diporocyclus*
*Tharo*	*Thalassiosira rotula*
*Tri*sp	*Trichodesmium* spp.
*Trifu*	*Tripos furca*
*Uln*sp	*Ulnaria* spp.
