# Supplementary figures and images for: Coupled Hydrological and Biogeochemical Forcings Structure Phytoplankton Community Assembly in a Eutrophic Estuary
Source: Microorganisms. 2026 Jun 18;14(6):1363. doi: 10.3390/microorganisms14061363 (PMC13304156; doi:10.3390/microorganisms14061363)

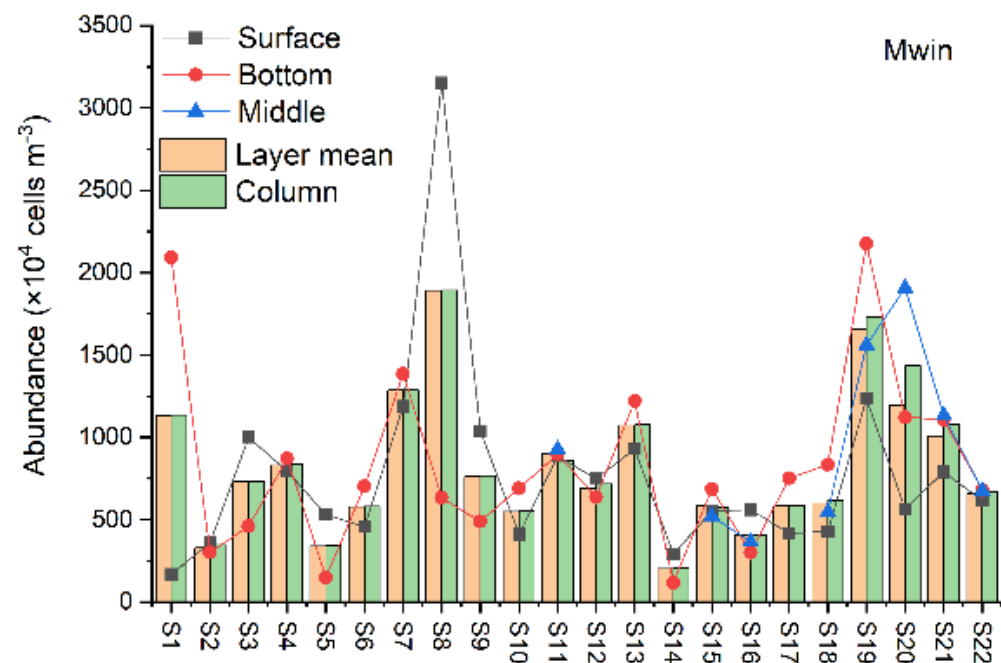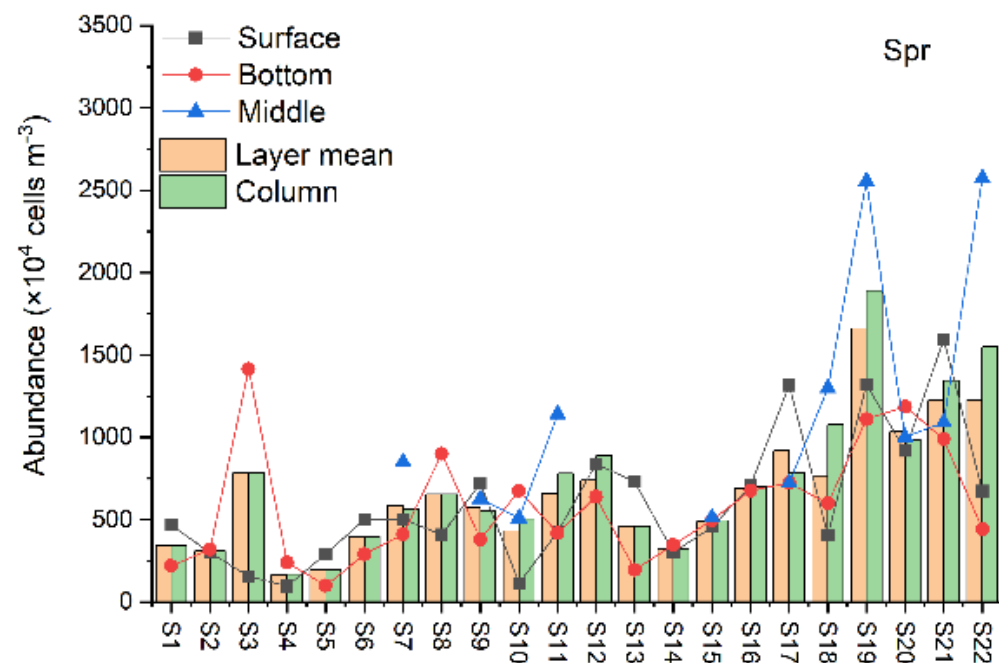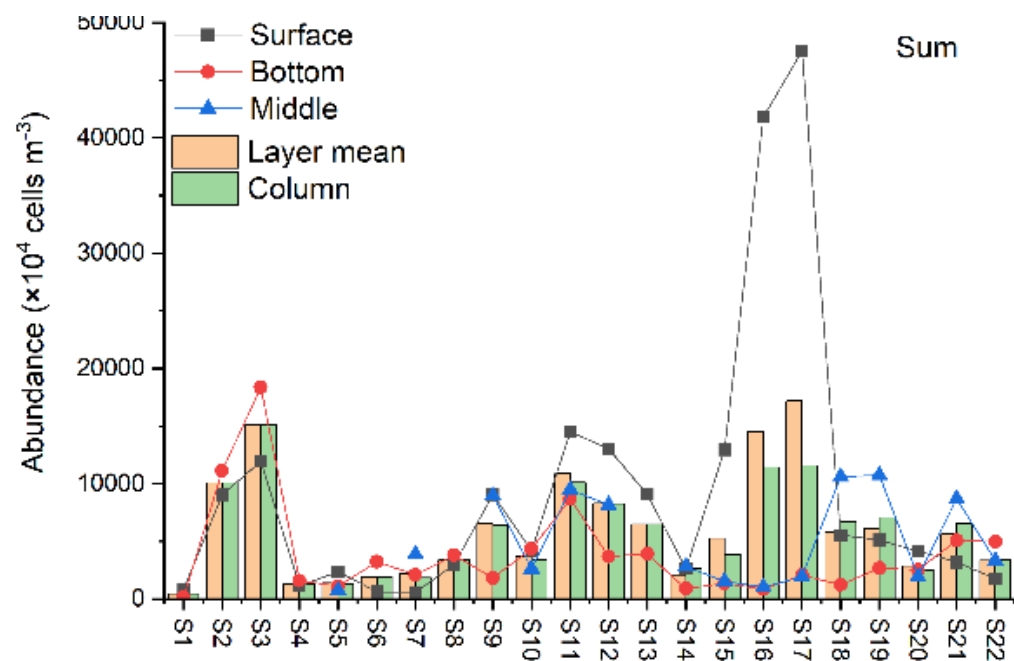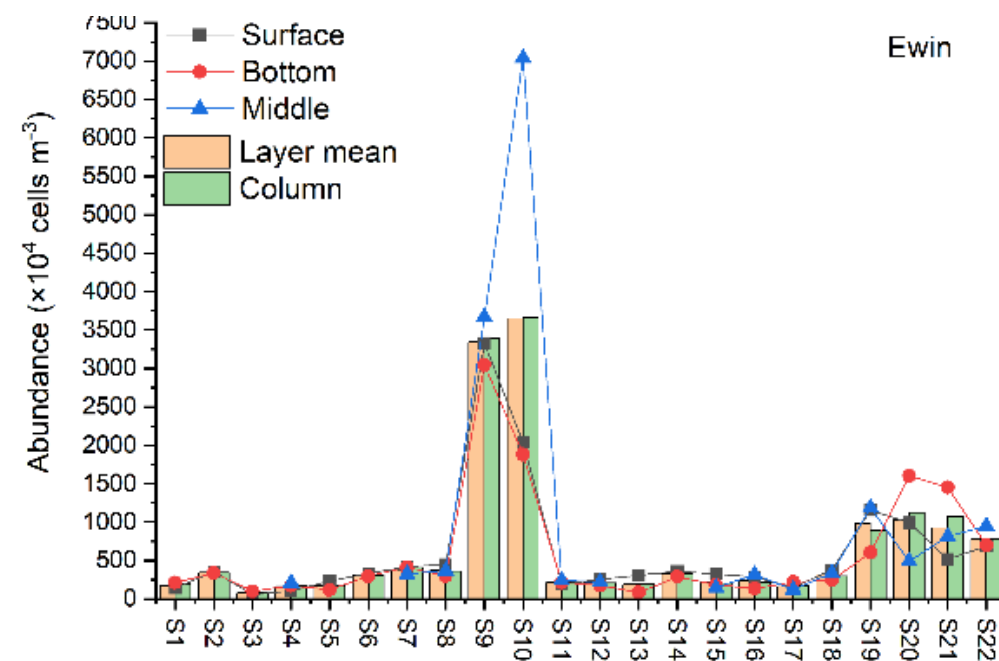

Supplement: Supplementary file 1 [file microorganisms-14-01363-s001.zip › Figure S1.pdf]

Mid-winter

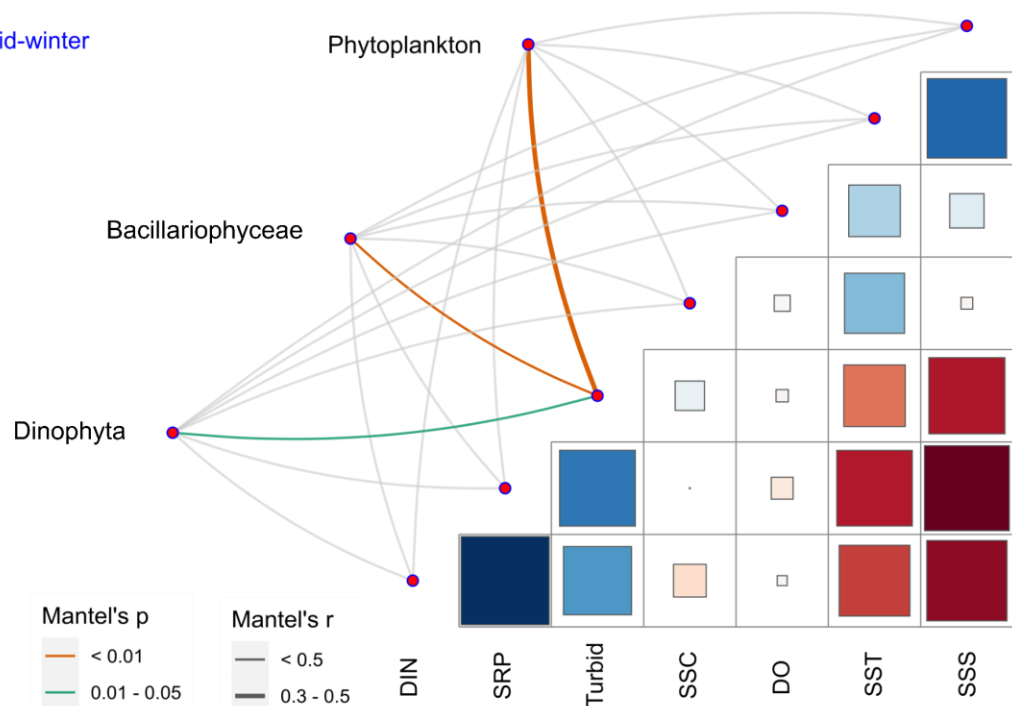

Spring

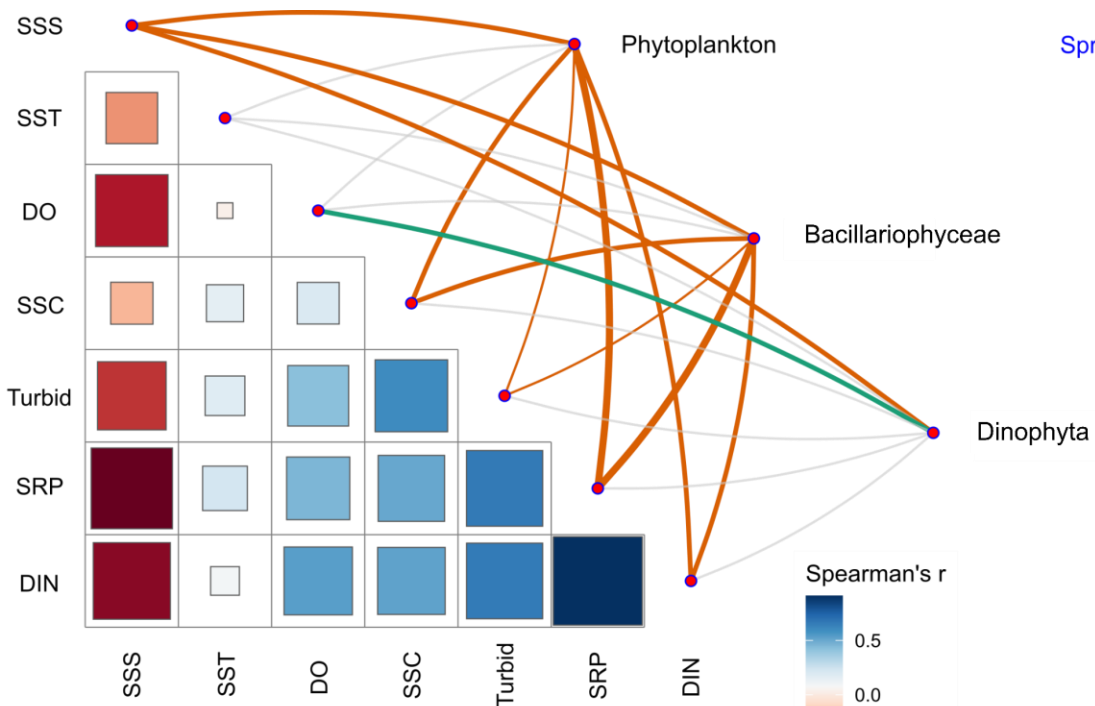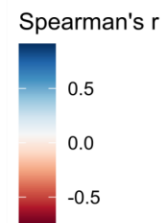

Summer

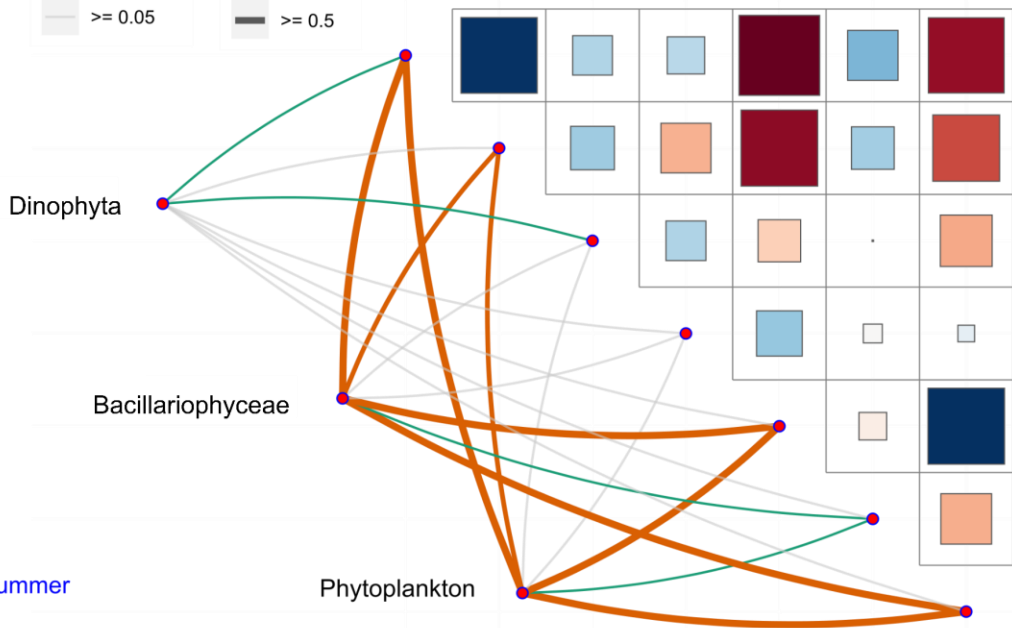

Early winter

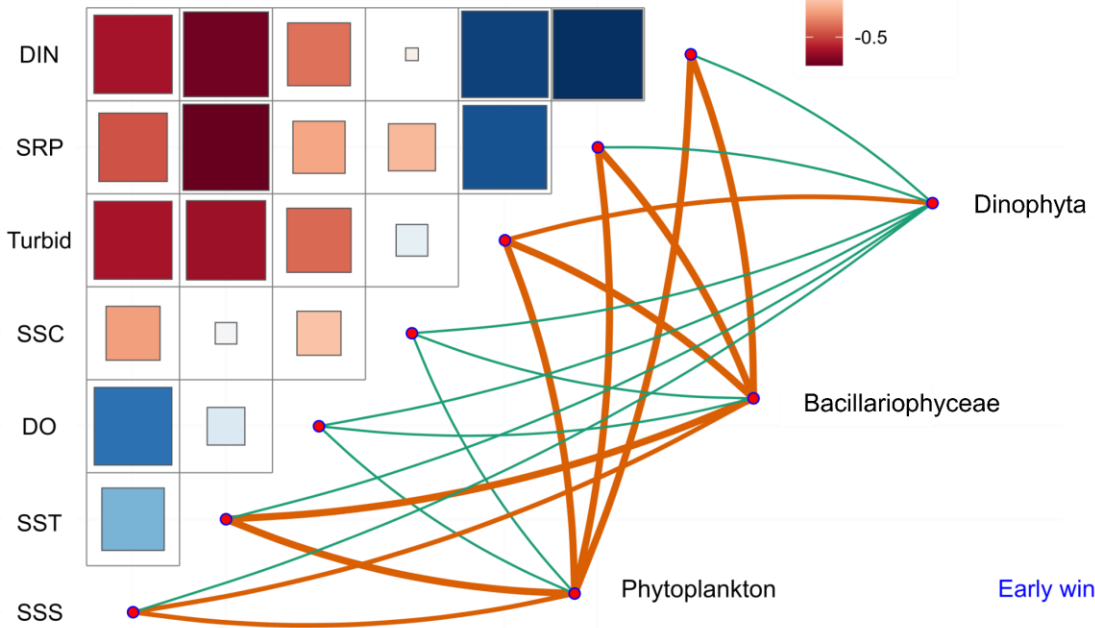

Supplement: Supplementary file 1 [file microorganisms-14-01363-s001.zip › Figure S2.pdf]
